# Pan-American Medical Congress

**Published:** 1896-08

**Authors:** 


### Society Notes.

### Rules for the Second Pan-American Medical Congress.

To meet in the City of Mexico, November 16, 17, 18 and 19, 1896.

Enrollment.—Article 1. In order to be properly enrolled, each member of the Congress will pay to the Treasurer thereof, in the City of Mexico, the sum of five dollars in gold.

General Sessions.—Article 2. There will be one opening session, one closing, and one intermediate session of a purely scientific character.

Art. 3. The opening session, which will be of a solemn character, and presided over by the Supreme authority of the Nation, besides being attended by the members of the Congress, will also be attended by the members of scientific societies, and other distinguished persons who may be invited. The session will be opened with the report of the General Secretary.

This will be followed by an address of welcome by the President of the Congress.

Two members will then speak on scientific subjects, and they will be followed by a speech from the President of the Republic.

Art. 4. At the closing session, the General Secretary will announce the place for holding the third meeting.

Sessions of the Sections.—Art. 9. The sessions will be held from 9 to 12 a. m., and from 3 to 5 p. m., in the places that may be designated by the Organizing Committee. They shall be presided over by the president of each section, alternating with the vice-presidents of each one o f the nations that are represented in the respective sections.

Papers, Extracts Thereof, and Discussions in the Sessions of the Sections.—Art. 15. All papers will be presented in writing.

Art. 16. Each author will forward to the secretary of the Organizing Committee, in the City of Mexico, and before the first day of August of the present year, an extract not exceeding 300 words, of the paper to be presented by him. These extracts will be printed in English, French and Spanish, and will be distributed to the members of the Congress, before the session in which they are to be read.

Art. 17. No paper will be announced which is not accompanied by this extract; but the authors who comply with these conditions, will have a right to have their work published intact in the transactions of the Congress.

Art. 18. The reading of the papers in the sessions must not last more than twenty minutes; when the papers are so long that they can not be read within that time, the authors will give extracts from them, either in writing or by speech; but they will be published intact in the transactions of the Congress, and in the language in which they have been written.

Art. 19. The extracts referred to in the preceding article will be delivered at the same time as the papers, to the secretary of the Section to which they pertain.

Art. 20. The members of the Congress who may take part in the discussions in any section, will present their speeches in writing, at the termination of the sessions, to the respective secretaries of such sections, and they will also be published in the transactions.

Art. 21. The papers which have been announced for reading in the order of the day in each Section, will serve as subjects for discussion. In such discussions no speaker will be allowed to speak more than once and for five minutes; but the author of the paper under discussion will be allowed to reply, if he considers it necessary, in one sole speech, which will not go beyond ten minutes.

Dr. Manuel Carmony y Valle,
Dr. Rafael Lavista,
Dr. Eduardo Liceaga.

---

### The Second Pan-American Medical Congress

The committee on organization of the second Pan-American Medical Congress has elected Dr. Manual Carmona y Valle President, Dr. Rafael Lavista Vice-President and Dr. Eduardo Liceaga Secretary, and has announced November 16, 17, 18, 19, 1896, as the date of the meeting to be held in the City of Mexico.

The most cordial invitation is extended to the medical profession of the United States to attend and participate in the meeting.

Titles of papers to be read should be sent at the earliest possible date to Dr. Eduardo Liceaga, Calla de San Andres, num 4, Ciudad de Mexico D. F. Republica Mexicana.

The date selected is in the midst of the delighful midwinter season, when the climate of Mexico is most attractive to the northern visitor.

The occasion should stimulate the medical profession of the United States to a most cordial reciprocation of the generous patronage accorded the Washington meeting of the Congress by our Mexican confreres.

It should be remembered that the United States is the largest, and in many regards the most important of the American countries, and that as a consequence more is expected of it than of any other Occidental nation. In no particular is this more true than in the maintenance of position in the realm of scientific medicine on the Western Hemisphere. It is, therefore, simply essential that in this Congress—the most important of all Medical Congresses, in its exclusive, yet broad, American significance—the best thought and the best work of the American profession shall be conspicuous in the proceedings.

The zeal and enthusiasm of the Mexican profession, and the active interest of the Mexican Government are co-operating to make the second Pan-American Medical Congress attractive, important and memorable.

Those who contemplate attending should send their names and addresses at as early a date as possible to Dr. Charles A. L. Reed, St. Ledger Place, Cincinnati, that the committee in Mexico may be advised of the probable attendance.

William Pepper,
Ex-Officio President.
A. M. Owen,
A. Vander Veer,
Charles A. L. Reed,
Ex-Officio Secretary.
International Executive Committee for the United States.

